# Differentiation between thyroid‐associated orbitopathy and Graves’ disease by iTRAQ‐based quantitative proteomic analysis

**DOI:** 10.1002/2211-5463.13172

**Published:** 2021-05-26

**Authors:** Jianshu Kang, Yunqin Li, Zhijian Zhao, Hong Zhang

**Affiliations:** ^1^ Department of Ophthalmology The Second People’s Hospital of Yunnan Province The Fourth Affiliated Hospital of Kunming Medical University China; ^2^ Yunnan Eye Institute Kunming China; ^3^ Key Laboratory of Yunnan Province for the Prevention and Treatment of Ophthalmologya Kunming China; ^4^ Yunnan Eye Disease Clinical Medical Center Kunming China; ^5^ Yunnan Eye Disease Clinical Medical Research Center Kunming China

**Keywords:** Graves’ disease, inflammatory response, iTRAQ technique, MYH11, proteomics, thyroid‐associated obitopathy

## Abstract

Graves’ ophthalmopathy, also known as thyroid‐associated orbitopathy (TAO), is the most common inflammatory eye disease in adults. The most common etiology for TAO is Graves’ disease (GD); however, proteomic research focusing on differences between GD and TAO is limited. This study aimed to identify differentially expressed proteins between thyroid‐associated orbitopathy (TAO) and GD. Furthermore, we sought to explore the pathogenesis of TAO and elucidate the differentiation process via specific markers. Serum samples of three patients with TAO, GD, and healthy controls, respectively, were collected. These samples were measured using the iTRAQ technique coupled with mass spectrometry. Differentially expressed proteins in TAO and GD were identified by proteomics; 3172 quantified proteins were identified. Compared with TAO, we identified 110 differential proteins (27 proteins were upregulated and 83 were downregulated). In addition, these differentially expressed proteins were closely associated with cellular processes, metabolic processes, macromolecular complexes, signal transduction, and the immune system. The corresponding functions were protein, calcium ion, and nucleic acid binding. Among the differential proteins, MYH11, P4HB, and C4A were markedly upregulated in TAO patients and have been reported to participate in apoptosis, autophagy, the inflammatory response, and the immune system. A protein–protein interaction network analysis was performed. Proteomics demonstrated valuable large‐scale protein‐related information for expounding the pathogenic mechanism underlying TAO. This research provides new insights and potential targets for studying GD with TAO.

## Graphical abstract

Serum samples were collected from patients with TAO and Graves’ disease (GD), and healthy controls (three cases in each group). After extraction and quantification, the obtained proteins were treated with ITRAQ reagent and then analyzed using LC‐MS/MS. Finally, the identified differentially expressed proteins in TAO and GD were subjected to bioinformatics analysis, including GO analysis, KEGG analysis, and PPI analysis.

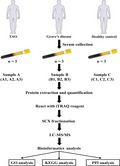

**Abbreviations**
CTLA: cytotoxic T lymphocyte‐associated antigen
GD: Graves’ disease
MYH11: myosin heavy chain 11
P4HB: poly‐4‐Hydroxybutyrate
TAO: thyroid‐associated orbitopathy

Graves’ ophthalmopathy, also known as thyroid‐associated orbitopathy (TAO), is the most common inflammatory eye disease in adults. TAO is an extra‐thyroidal manifestation of autoimmune thyroid disease; 25%–50% of patients with Graves’ disease (GD) develop TAO without any known predictive factor [1]. TAO has distinct clinical features, including eyelid retraction, restrictive strabismus, proptosis, and some TAO patients are at risk for losing their sight. The risk factors for TAO include cigarette smoking and genetic factors [2]. Thyroid‐stimulating hormone‐receptor (TSH‐R) antibodies (TSH‐R‐Abs) are detectable in patients with TAO. TSH‐R levels are associated with TAO activity and severity. Nevertheless, TSH‐R‐Abs are not detectable in all patients [3]. TAO can cause acute inflammatory events, so pro‐inflammatory cytokines, such as IL‐6, IL‐22, IL‐17, and C‐C chemokine ligand 20 (CCL20), are subject to positive regulation. Thus far, many gene factors have been confirmed to have an association with TAO, including cytotoxic T lymphocyte‐associated antigen‐4 (CTLA‐4), an HLA class II molecule (HLA‐DRB‐1), and TNF‐α [4]. Recent reports suggest that CD4+T cells (Th1, Th2, and Th17), CD5+B cells, and bone marrow‐derived CD34+ fibrocytes contribute to the pathogenesis of TAO [4, 5].

Proteomics analysis has been shown to be an effective approach to assess the differential proteins in patients with autoimmune thyroid diseases on a large scale [6, 7, 8]. Mass spectrometry‐based quantitative proteomics is a powerful method for gene screening and evaluation of regulated biological processes on a functional level, ultimately reflecting the pathologic conditions. Currently, proteomics profiles of tears and orbital tissues from TAO patients differ from healthy controls based on matrix‐assisted laser desorption/ionization time‐of‐flight mass spectrometry technology (MALDI‐TOF MS) [9]. These studies reported specific altering proteins in TAO patients, such as H4, POTEE, AOC3, LYZ, and MYH3 [9]. These proteins are involved in biological processes, such as cell proliferation, apoptosis, inflammatory responses, immune responses, and endoplasmic reticulum stress [6, 7]. The finding that proteins are overexpressed in TAO patients provided a molecular basis for the pathogenesis of TAO [9]. The most common etiology for TAO is GD; however, proteomic research focusing on differences between GD and TAO is limited [10].

## Materials and methods

### Participants

Nine participants were included in this study. Three patients with TAO had a clinical activity score (CAS) > 4, three patients had GD, and there were three healthy controls. The TAO CAS is comprised of 10 items [9]. In the current study, all patients with GD were diagnosed based on thyroid ultrasonography, a thyroid hormone assay, orbital CT, and other auxiliary examinations; radiotherapy, chemotherapy, targeted therapy, or immunotherapy was not performed. In addition, patients with similar ocular symptoms were excluded.

### Serum sample collection

All patients and controls underwent peripheral blood collection. The blood samples were centrifuged to separate the serum, which was stored at −80 °C. The serum levels of anti‐thyroid‐stimulating antibody (TSAB), anti‐thyroglobulin antibody (TGAB), and anti‐thyroid peroxidase antibody (TPOAB) were measured in all patients.

### Protein extraction and quantification

The serum samples were removed from high‐abundance proteins using the ProteoMinerTM protein enrichment kit (Bio‐Rad, Shanghai, China), and the protein concentration of the treated serum was determined by a Bradford protein quantitative kit (Beyotime, Shanghai, China) according to the manufacturer’s instructions.

### iTRAQ experiment and LC‐MS/MS analysis

SDS/PAGE was used to identify the integrity of the proteins prior to follow‐up experiments. A total of 20 μg of the protein sample was separated by 12% SDS/PAGE and stained. The proteins were collected according to the following protocol: digestion with trypsin; desalting by gel filtration; thrice‐washing with PBS; elution; and lyophilization, as previously described [11]. Then, the iTRAQ reagent (Beyotime, Shanghai, China) was used to label the proteins according to the manufacturer’s protocol.

The labeled peptides were graded using the Rigol L3000 HPLC system (Dalian, China) and a Waters BEH C18 column (4.6 × 250 mm, 5 μm; (Dalian, China). The details of the elution gradient are shown in Table S1. Proteomics analyses were performed using an EASY‐nLCTM 1200 UHPLC system (Thermo Fisher, Waltham, MA, USA) coupled with a Q Exactive HF‐X mass spectrometer (Thermo Fisher) using a linear gradient elution, as listed in Table S2. The separated peptides were analyzed using a Q Exactive HF‐X mass spectrometer (Thermo Fisher).

### Database search and data interpretation

The LC‐MS/MS data were processed and searched against the UNIPORT *Homo sapiens* protein sequence database by Proteome Discoverer 2.2 (Thermo Fisher). Variable modifications consisted of oxidation of methionine (M), acetylation of the N terminus, and iTRAQ 8‐plex of tyrosine and lysine, as specified in PD 2.2.

The data were calculated by a decoy database searching for false discovery rate (FDR) analysis. The FDR was set to 1%. The peptide‐labeled iTRAQ 8‐plex was selected as the quantification mode. The protein quantification results were statistically analyzed with the Mann–Whitney test. Multiple testing corrections were used to adjust *p*‐values to control the FDR. Only proteins with a fold change > 1.2 and adjusted *P*‐values < 0.05 were considered to be differentially expressed proteins (DEPs).

### Bioinformatics

The Gene Ontology (GO) and InterPro (IPR) analyses were performed using the Interproscan‐5 program (https://www.cnblogs.com/wq242424/p/4701968.html) against a nonredundant protein database, including Pfam, PRINTS, ProDom, SMART, ProSiteProfiles, and PANTHER [12]. The Kyoto Encyclopedia of Genes and Genomes (KEGG) was used to categorize the protein family and pathway. The probable protein–protein interactions were predicted using the STRING‐DB server (http://string.embl.de/) [12] and visualized with Cytoscape software (San Francisco, CA, USA). The enrichment pipeline [13] was used for enrichment analysis of GO, IPR, and KEGG. It has been reported that hyperthyroidism is associated with the development of orbital disease [14]. The number of upregulated proteins (C vs. A group and C vs. B group) was significantly greater than the upregulated proteins (B vs. A group).

### Ethics approval and consent to participate

This study was approved by the Second People’s Hospital of Yunnan Province Ethics committee (Ethical Review Number: 2019173). The procedures in the present study were all performed following the ethical standards of the responsible Committee on Human Experimentation and with the Helsinki Declaration revised in 2008. Informed written consent was obtained from all participating subjects.

## Results

### Demographic data of the patients and healthy controls

The clinical characteristics of TAO patients, GD patients, and healthy controls are shown in Table 1. The three TAO patients (2 males and 1 female) had an age range of 42–54 years. The three GD patients (1 male and 2 females) had an age range of 34–50 years. The 3 healthy controls had an average age of 26 years. Two TAO patients and one GD patients smoked cigarettes. All TAO patients were TPOAB‐positive and had a CAS > 4. None of the TAO patients had received treatment.

**Table 1:** Clinical characteristics of participants.

Groups	TAO	Graves' disease	Healthy controls
Number	3	3	3
Male/Female	2/1	1/2	0/3
Age range	42–54	34–50	25–27
Smokers	2	1	0
TPOAB	Positive	/	/
CAS value	>4	/	/

### Proteomics analysis

**Fig. 1:**
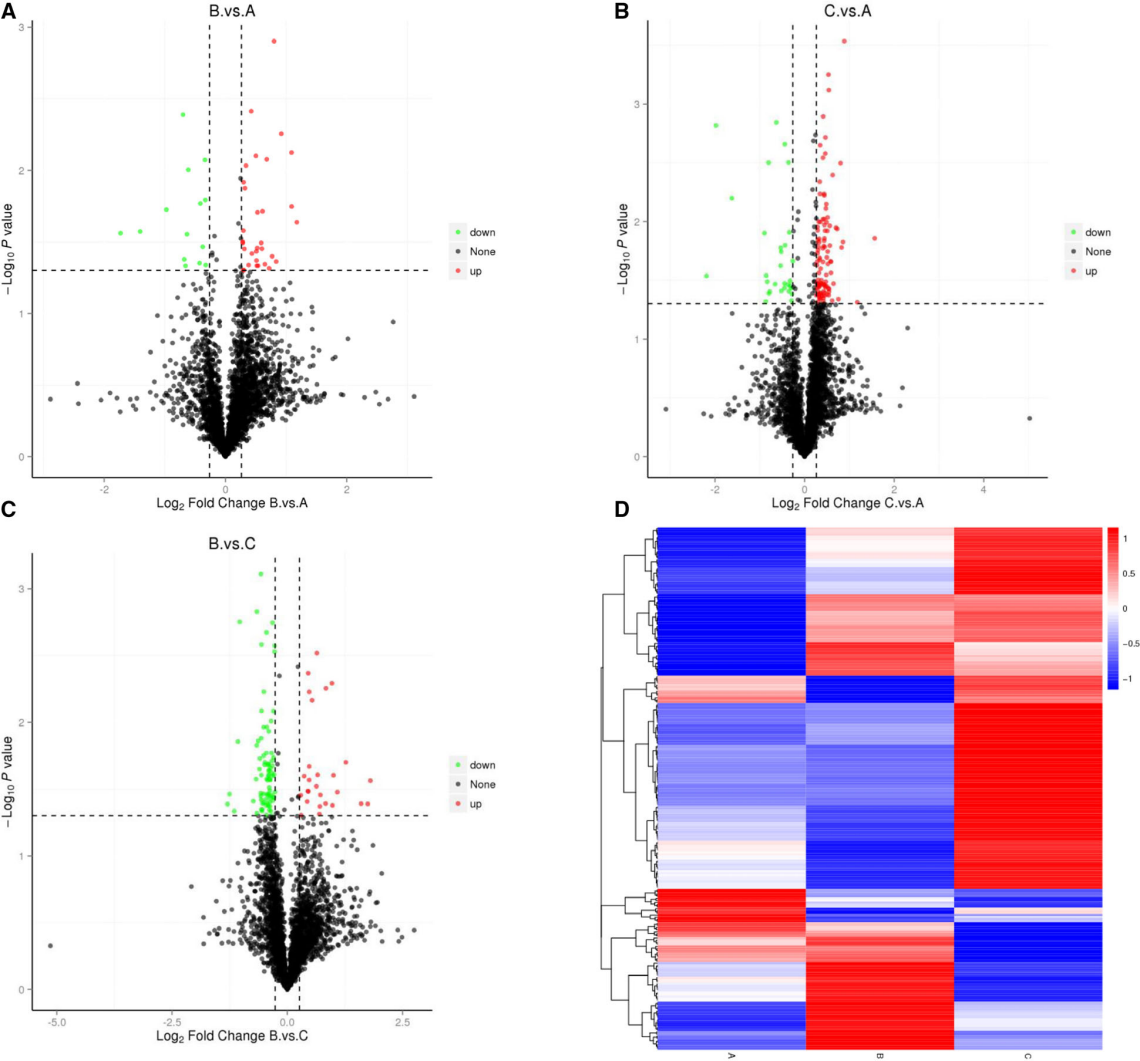
Differentially expressed proteins are displayed with volcanic maps (A–C) and heatmaps (D). (A–C) The *x*‐axis represents multiple differences in different proteins (annotated by the log2 value), the *y*‐axis represents the *P*‐value (annotated by the −log10 value). The black points represent the proteins with nonsignificant differences, the red points represent the upregulated proteins, and the green points represent the downregulated proteins. B vs. A is the group of Graves’ disease vs. healthy controls (A); C vs. A is the group of TAO patients vs. healthy controls (B); and B vs. C is the group of Graves’ disease vs. TAO patients (C). (D) The cluster heatmap was applied to observe the upregulated and downregulated proteins in different samples. The red modules indicate increased proteins, and the blue modules indicate decreased proteins. Significant proteins were those passing the two tests at a cutoff of false discovery rate (FDR) < 0.05. Plot_transcript_heatmap function in Sleuth package was utilized to visualize the cluster analysis. EnhancedVolcano R package was used to generate the volcano plot.

Knowledge of changes in protein expression among TAO and GD patients or healthy controls helps to understand the pathogenesis of TAO, make an early diagnosis, and prevent progression. In this study, we used iTRAQ coupled with LC‐MS/MS to identify 3172 proteins in the 3 study groups (healthy controls, A group; GD patients, B group; and TAO patients, C group; Table 2). Based on the MS data, a 1.2‐fold change in the cutoff value and a *P*‐value < 0.05 for the 3127 proteins were used as the screening criteria. We found that a total of 46 proteins had a significant change (*P* < 0.05) in the levels of expression between the B and A groups (Table 2). A total of 110 proteins had a significant change (*P* < 0.05) in the levels of expression between the B and C groups. A total of 136 proteins exhibited a significant change (*P* < 0.05) in the levels of expression between the C and A groups. Thirty‐two proteins were upregulated, and 14 proteins were downregulated based on a comparison of the GD patients and the healthy controls. Eighty‐three proteins were downregulated, and 27 proteins were upregulated in the GD patients compared to the TAO patients. An abundance of proteins had dysregulated levels of expression (106 upregulated and 36 downregulated) in TAO patients compared with the levels of expression in healthy controls. A volcano map was created, in which the red (upregulated) and green points (downregulated) had differential data points (Fig. 1A‐C). Each point represents a protein in the volcano map. The number of upregulated proteins (C vs. A group and C vs. B group) was significantly greater than the upregulated proteins (B vs. A group) based on the volcano map. The results were more intuitive in the heatmap in which most proteins were upregulated in TAO patients (Fig. 1D). The details of the MS data are shown in Tables S3‐S6. Specifically, Table S5 shows that prolactin‐inducible protein (PIP) and apolipoprotein F (APOF) analogues, two important proteins that regulate the immune response in humans, were significantly upregulated in TAO vs. GD.

**Table 2:** An overview of proteins quantified in this study. Study groups: healthy controls (A), Graves’ disease (B), TAO (C). In total, 3172 proteins were quantified. The Mann–Whitney test was applied to analyze the significantly regulated proteins. Proteins with a fold change > 1.2 and adjusted *P*‐values < 0.05 were considered to be differentially expressed proteins (DEPs).

Compared sample	Num. of total Quant.	Regulated type	Fold change > 1.2	Fold change > 1.3	Fold change > 1.5	Fold change > 2.0
B vs. A	3172	upregulated	32	23	13	3
		downregulated	14	10	8	2
B vs. C	3172	upregulated	27	24	16	6
		downregulated	83	59	13	5
C vs. A	3172	upregulated	106	62	14	2
		downregulated	30	20	12	3

### GO classification of DEPs

**Fig. 2:**
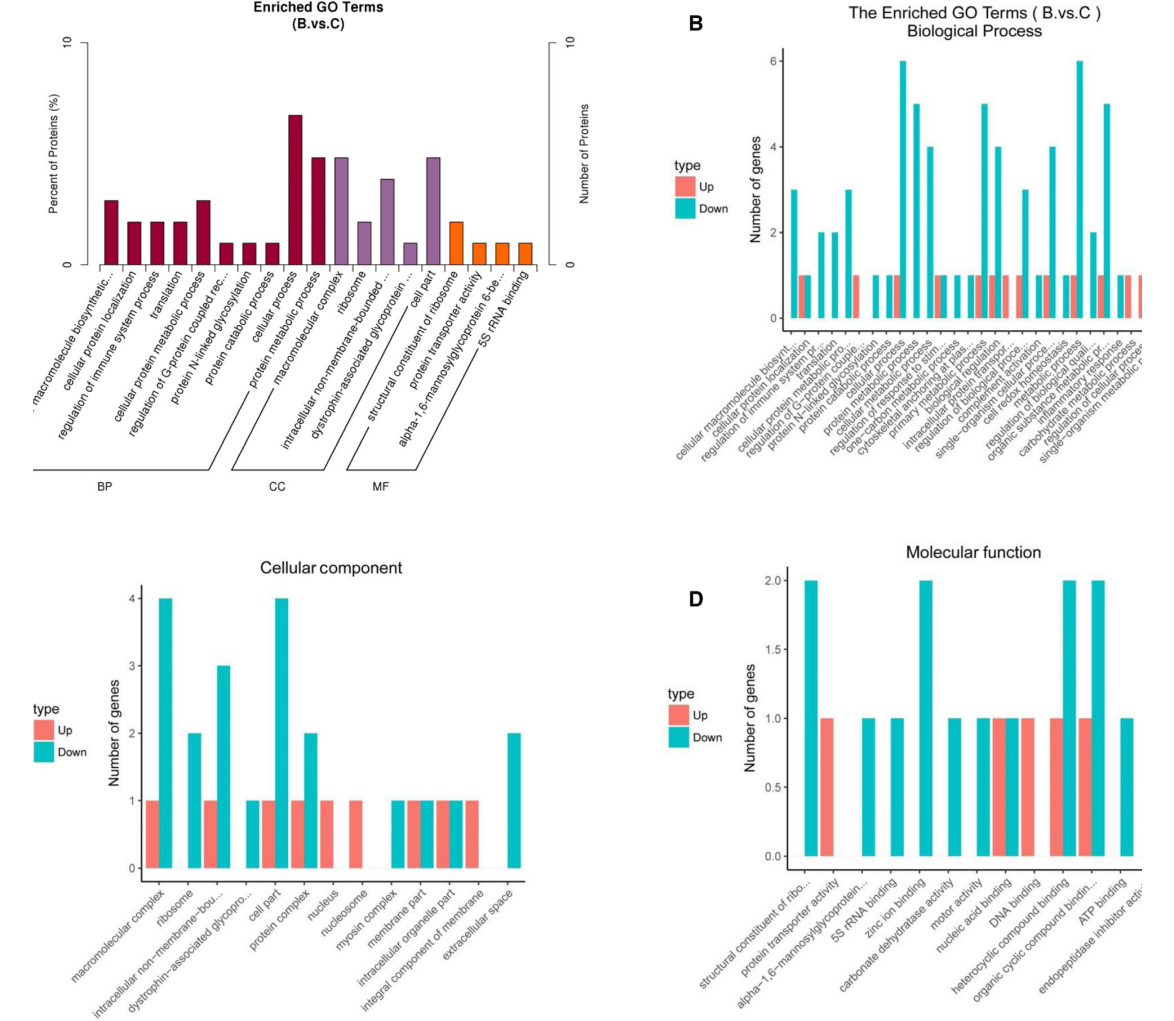
Gene Ontology (GO) annotation of the differentially accumulated expressed proteins in Graves’ disease vs. TAO patients. (A) The total of the differentially expressed proteins was evaluated by biological processes (BPs), cellular components (CCs), and molecular function (MF) terms. The *x*‐axis represents the GO categories, and the *y*‐axis represents the number of proteins. (B–D) The Enriched GO terms of upregulated and downregulated proteins were respectively represented with BPs (B), CCs, and MF terms (D). The types of dysregulated proteins are annotated with red (upregulated proteins) and green (downregulated proteins).

To further obtain an understanding of the functional classification of DEPs, GO analysis was performed. In our work, the 83 downregulated and 27 upregulated proteins were subjected to PANTHER for GO analysis. The several significantly enriched biological processes (BPs) are detailed, including the cellular component (CC) and molecular function (MF) terms, in Fig. 2A. As shown in Fig. 2B, the three largest groups of downregulated proteins were involved in cellular, metabolic, and organic substance metabolic processes. In addition, a minority of the downregulated proteins shown in Fig. 2B were involved in the immune system and inflammatory processes. Cellular parts and macromolecular complexes were found for the CC of most downregulated proteins (Fig. 2C). Zinc ion, organic cyclic compound, and heterocyclic compound binding and structural constituents of ribosomes were involved in the molecular function of most downregulated proteins (Fig. 2D). A few upregulated proteins were involved in DNA binding and protein transporter activity. The details of the GO enrichment analysis involving B vs. A groups and C vs. A groups are shown in Figs S1 and S2.

### Biological functions by KEGG analyses

**Fig. 3:**
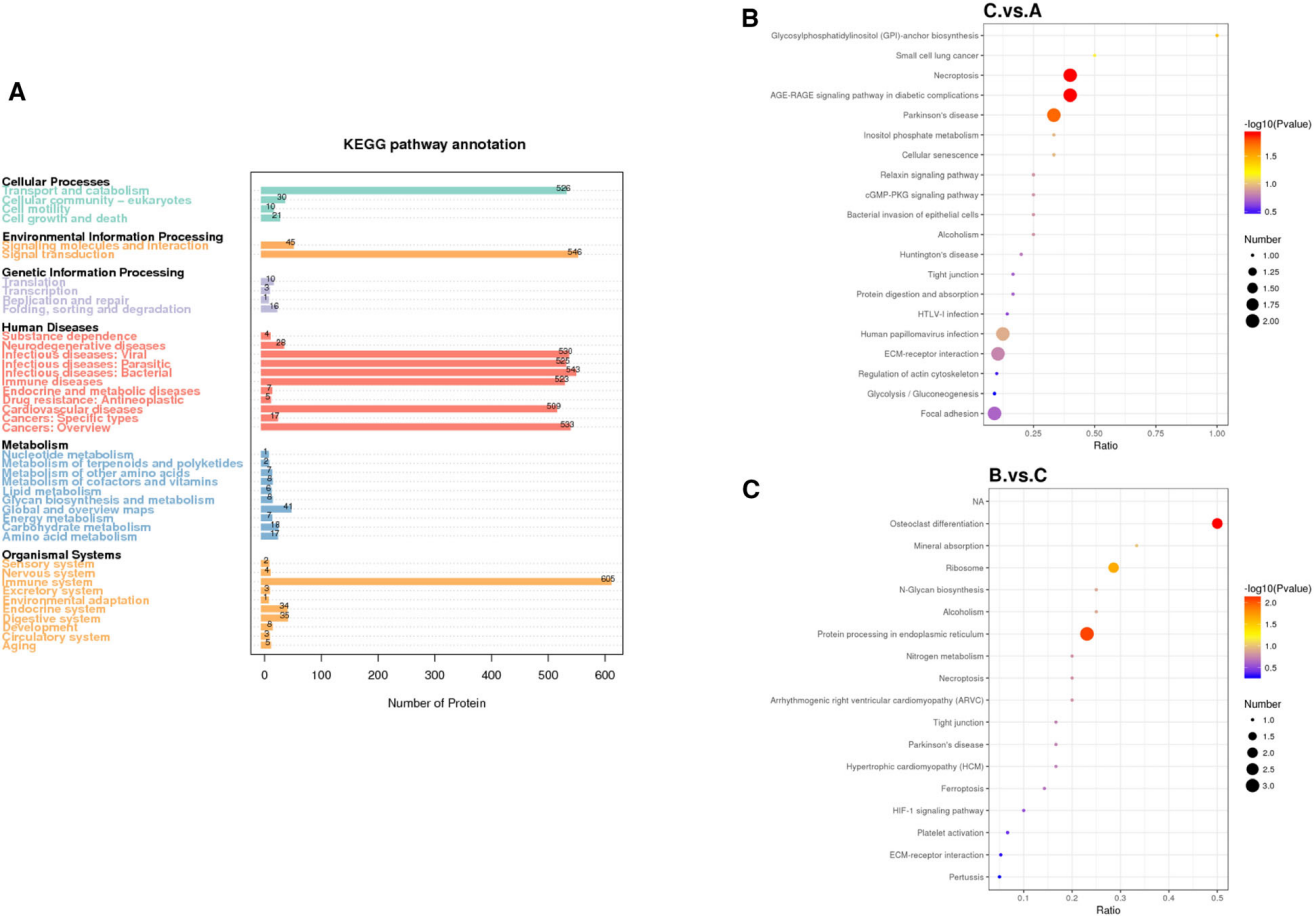
KEGG analysis of the differentially expressed proteins. (A) An overview of the identified proteins in the three groups of samples was constructed by KEGG pathway analysis. (B) KEGG analysis of the differentially expressed proteins following comparison of TAO patients vs. healthy controls (C vs. A). (C) KEGG analysis of the differentially expressed proteins following comparison of Graves’ disease vs. TAO patients (B vs. C). The size of the points represents the number of differential proteins in the correlative pathway. The color of the dots ranges from blue‐to‐red, representing the *P*‐value. The deeper red dots represent greater statistical significance.

KEGG pathway enrichment analysis was used to identify the major biochemical metabolic and signal transduction pathways of the DEPs. As shown in Fig. 3A, an overview of 3169 identified proteins in the 3 groups of samples (detailed information in Table S7) revealed a mass of proteins based on KEGG annotation that were distributed in transport and catabolism, signal transduction, immunologic diseases, and infectious diseases. The 29 DEPs were analyzed by KEGG annotation in the C vs. A groups. The top four enriched pathways were glycosylphosphatidylinositol (GPI)‐anchor biosynthesis, small cell lung cancer, necroptosis, and the AGE‐RAGE signaling pathway in diabetic complications (Fig. 3B and detailed information in Table S8). The 27 DEPs among the B vs. C groups based on KEGG annotation were significantly enriched in osteoclast differentiation, mineral absorption, ribosomes, and N‐glycan biosynthesis as the top four enriched pathways (Fig. 3C and detailed information in Table S9).

### Protein–protein interaction analysis of the most overrepresented processes

**Fig. 4:**
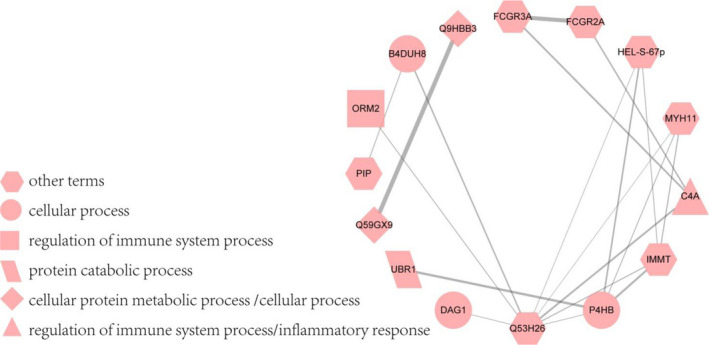
Protein–protein interaction network of regulated proteins in Graves’ disease vs. TAO patients. The proteins involved in the main processes were shown with different geometries, including cellular, protein catabolic, cellular protein metabolic, and regulation of immune system processes, and the inflammatory response. The proteins were noted with gene name, except that some other proteins were noted with protein ID, as follows: Q59GX9; Q9HBB3; Q53H26; and B4DUH8. The relative proteins were ribosomal protein L5 variant, 60S ribosomal protein L6, transferrin variant, and carbonic anhydrase 6 analogue. These proteins were all upregulated in TAO compared with Graves’ disease patients.

Furthermore, a protein–protein interaction network analysis for the significantly enriched process from GO analysis was performed using the STRING website. The resulting network was visualized by Cytoscape software (Fig. 4 and detailed information in Table S10). A number of proteins involved in metabolic, cellular, protein catabolic, and regulation of immune system processes, and the inflammatory response were of great interest with respect to protein expression of the B vs. C groups. A significant network map was displayed covering 15 proteins and 19 distinct interactions (Fig. 4). As shown in Fig. 4, different geometries represented different processes, and some proteins were involved in multiple processes.

## Discussion

iTRAQ quantitative proteomic technology has been used to identify specific proteins in patients and reveal the mechanisms underlying diseases. In our study, 3172 proteins were profiled, of which 46 showed a significant change in GD patients vs. the healthy control group, 110 proteins showed a significant change in GD vs. TAO patients, and 136 proteins exhibited a significant change in TAO patients vs. the healthy control group. More upregulated proteins were identified in TAO vs. GD patients and TAO patients vs. healthy controls compared to the downregulated proteins. Therefore, a more vigorous inflammatory response occurred in TAO patients, which is consistent with previous studies involving proteomic analyses of orbital tissues and tears in TAO patients [6, 7]. In addition, for the first time, we used the iTRAQ technique combined with LC‐MS/MS to identify the changed proteins in the serum of TAO and GD patients or healthy controls. A total of 3172 proteins in 3 groups of samples were quantified and showed significant regulation, of which 110 were in the GD vs. TAO groups, 136 in the TAO patients vs. healthy controls, and 46 in the GD patients vs. healthy controls. These alternative proteins may participate in cellular and metabolic processes, and the immune system. Our study may provide an overview of protein expression patterns in TAO patients. Moreover, our study could help to provide an in‐depth understanding of the pathogenesis of TAO. The findings are hoped to facilitate making an early diagnosis of TAO and preventing GD from developing into TAO or more severe disease.

Based on GO analysis, the results of the comparison among the three groups (TAO, GD, and healthy controls) further revealed that protein metabolic processes and inflammatory progression were more vibrant in patients with TAO. Based on KEGG analysis, we found that many DEPs were involved in a signaling pathway, including osteoclast differentiation, ribosome, and protein processing in the endoplasmic reticulum. The upregulated FCGR2A and FCGR3A are immunoglobulin gamma Fc receptor variants. Barrera et al. [14] reported that FCGR2A and FCGR3A might be more susceptible to rheumatoid arthritis (RA), which implied that a vigorous inflammatory response might induce an increase in Fc gamma receptors, which is in agreement with a previous study that reported proteins involved in inflammatory processes are upregulated in TAO [8]. Moreover, ribosomes are essential for protein synthesis, and ribosomal proteins are devoted to cell proliferation, differentiation, and apoptosis [15]. Our data showed that some ribosomal proteins, such as the 60S ribosomal protein, L6, and the ribosomal protein, L5, were upregulated in TAO vs. GD patients. A previous study reported that proteins involved in apoptosis are overexpressed in TAO [6]. Some proteins processed in the endoplasmic reticulum are upregulated in TAO vs. GD patients [16]. Cytoskeleton‐associated protein 4 (CKAP4) is an endoplasmic reticulum protein that regulates cell migration and apoptosis. Fei et al. [16] found that CKAP4 induces apoptosis. UBR1, an E3 ubiquitin ligase, plays a role in the N‐end rule degradation pathway and downregulates the mTOR pathway [17]. Because the mTOR pathway mediates apoptosis and autophagy, UBR1 may be involved in the pathogenic mechanism underlying TAO. This finding is in agreement with the reports that the upregulation of specific proteins in orbital tissues from TAO was associated with cell proliferation, apoptosis, and endoplasmic reticulum stress [6].

Compared with previous studies, the proteomics of orbital tissues in TAO revealed that many myosins are significantly upregulated, [6] such as MYH2 (3.5‐fold), MYH6 (2.5‐fold), and MYH3 (2.3‐fold), as well as myosin heavy chain 11 (MYH11) in our proteomic data. In addition, several upregulated proteins in TAO vs. GD patients drew our attention, such as PIP and APOF analogues. It has been reported that PIP is critical for optimal CD4+Th1 cell differentiation, and PIP deficiency leads to impaired Th1‐mediated immune responses and IFN‐γ production [18]. Th1 cells induce the secretion of IL‐1β, IL‐2, IFN‐γ, and TNF‐α [4], which can lead to the development of TAO [19]. APOF is a component of the HDL and LDL fractions in human serum. The expression of APOF affects the IFN‐α‐induced gene levels associated with autoimmune diseases [20]. Further studies on these regulated proteins may help us elucidate the pathogenesis of TAO. The MS data of GD patients vs. healthy controls and TAO patients with healthy controls exposed some identical downregulated proteins, such as α‐2 macroglobulin (A2M) and cerebellin‐4 (CBLN4), which was similar to downregulated β2‐microglobulin based on proteomics of tear fluid in TAO patients [9]. Specifically, decreased β2‐microglobulin causes the upregulation of β2‐microglobulin‐free MHC class I molecules in TAO patients, thereby contributing to T‐cell activation [9]. A2M is a zinc‐binding protein and has a pivotal role in immune efficiency [21]. CBLN4 interacts with Netrin‐1, which has been shown to modulate the immune response by promoting CD4+T‐cell migration [22, 23]. It is likely that these downregulated proteins may be influenced by inflammatory processes. Furthermore, some GD patients with no apparent clinical GO have signs of orbital inflammation from infrared imaging [7]. The two downregulated proteins in patients with TAO and GD may provide a fresh perspective on the commonality between TAO and GD. In‐depth analyses of these proteomic data in the three groups were conducive to identify protein regulations of the disease, particularly the similarities and differences between GD and TAO.

Those DEPs interacted with other proteins that appeared to play essential roles in GD patients developing TAO. Combined with the MS data, several proteins were randomly selected, which were MYH11, Poly‐4‐Hydroxybutyrate (P4HB), and C4A. The proteins were all downregulated in the B vs. C groups. The three proteins were elevated in TAO patients compared with GD patients. Myosin heavy chain protein 11 (MYH11) is a major contractile protein in smooth muscle cells. It has been reported that myosins may be related to cell migration and adhesion, signal transduction, tumor suppression, and intracellular transport [24]. Recent studies have reported that myosins play essential roles in cancers [25, 26]. Low expression of MYH11 is correlated with colorectal, breast, and nonsmall‐cell lung cancer [27, 28, 29]. Nevertheless, Li et al. [28] found that MYH11 is upregulated in differentiated thyroid carcinoma by proteomic analysis using the iTRAQ technique. It has been reported that a risk factor for differentiated thyroid carcinoma is GD [30], suggesting that the level of MYH11 may be a prognostic marker for TAO. P4HB is an autophagy‐related gene and plays an important role in the endoplasmic reticulum. Autophagy is a critical homeostasis process in eukaryotes. Endoplasmic reticulum chaperones have been shown to be critical in regulating proliferation, apoptosis, and immunity [31]. GO analysis has demonstrated that P4HB is involved in the cell redox homeostasis process [32]. Protein disulfide‐isomerase (P4HB) has been upregulated in many cancer cell types [33]. Some studies have reported that P4HB facilitates peptide selection by MHC class I during antigen processing and promotes IFN‐γ production of immune cells [34]. The upregulation of P4HB may be a risk factor for inflammatory‐induced dysfunction diseases, such as TAO. C4A is a small protein derived from complement component C4 that is involved in innate immune surveillance. C4A is devoted to ERK activation through protease‐activated receptor (PAR)1 and PAR4 in a Gαi‐independent signaling pathway [35]. Indeed, fine‐tuning PAR1 signaling could be used to treat cardiovascular and inflammatory diseases [36]. C4 is upregulated in autoimmune disorders, such as rheumatoid arthritis (RA) [37]. An inflammatory response is one of the risk factors that can lead to TAO. A previous study has reported that proteins, which are involved in inflammatory processes, are upregulated in tear fluids from TAO patients [7].

In conclusion, our study systematically identified DEPs in patients with TAO, GD, and healthy controls. Most of the DEPs were involved in metabolism, inflammation, macromolecule biosynthesis, and single‐organism cellular processes [38, 39]. In addition, some of these altering proteins were investigated to illuminate the functional significance of the pathogenic mechanism underlying TAO. Taken together with previous studies, the present study may further help us to distinguish GD patients who later develop TAO from those who do not.

## Conflict of interest

We all declare that we have no conflicts of interest.

## Author contributions

All authors contributed to the study conception and design. Material preparation, data collection, and analyses were performed by JK, YL, and ZZ. The first draft of the manuscript was written by HZ, and all authors commented on previous versions of the manuscript. All authors read and approved the final manuscript.

## Supporting information

Fig S1. The details of the GO enrichment analysis involving B vs. A groups.
Fig S2. The details of the GO enrichment analysis involving C vs. A group.
Table S1. Peptide fraction separation liquid chromatography elution gradient table.
Table S2. Liquid chromatography elution gradient table.
Table S3. All differentially expressed proteins identified by iTRAQ in A vs. B groups, A vs. C groups, and B vs. C groups. A: Healthy controls, B: Graves’ disease, C: TAO.
Table S4. The differentially expressed proteins identified by iTRAQ in A vs. B groups. A: Healthy controls, B: Graves’ disease.
Table S5. The differentially expressed proteins identified by iTRAQ in B vs. C groups. B: Graves’ disease, C: TAO.
Table S6. The differentially expressed proteins identified by iTRAQ in A vs. C groups. A: Healthy controls, C: TAO.
Table S7. The KEGG analysis of all differentially expressed proteins in A vs. B groups, A vs. C groups, and B vs. C groups. A: Healthy controls, B: Graves’ disease, C: TAO.
Table S8. The KEGG analysis of the differentially expressed proteins in A vs. C groups. A: Healthy controls, C: TAO.
Table S9. The KEGG analysis of the differentially expressed proteins in B vs. C groups. B: Graves’ disease, C: TAO.
Table S10. The PPI analysis of the differentially expressed proteins in B vs. C groups. B: Graves’ disease, C: TAO.

## Data Availability

The datasets used or analyzed during the current study are available from the corresponding author upon reasonable request.
