# Supplementary material for: Differentiation between thyroid‐associated orbitopathy and Graves’ disease by iTRAQ‐based quantitative proteomic analysis
Source: FEBS Open Bio. 2021 May 26;11(7):1930–40. doi: 10.1002/2211-5463.13172 (PMC8255837; doi:10.1002/2211-5463.13172)
Supplement: Supplementary file 1 — Fig S1. The details of the GO enrichment analysis involving B vs. A groups. Fig S2. The details of the GO enrichment analysis involving C vs. A group. Table S1. Peptide fraction separation liquid chromatography elution gradient table. Table S2. Liquid chromatography elution gradient table. Table S3. All differentially expressed proteins identified by iTRAQ in A vs. B groups, A vs. C groups, and B vs. C groups. A: Healthy controls, B: Graves’ disease, C: TAO. Table S4. The differentially expressed proteins identified by iTRAQ in A vs. B groups. A: Healthy controls, B: Graves’ disease. Table S5. The differentially expressed proteins identified by iTRAQ in B vs. C groups. B: Graves’ disease, C: TAO. Table S6. The differentially expressed proteins identified by iTRAQ in A vs. C groups. A: Healthy controls, C: TAO. Table S7. The KEGG analysis of all differentially expressed proteins in A vs. B groups, A vs. C groups, and B vs. C groups. A: Healthy controls, B: Graves’ disease, C: TAO. Table S8. The KEGG analysis of the differentially expressed proteins in A vs. C groups. A: Healthy controls, C: TAO. Table S9. The KEGG analysis of the differentially expressed proteins in B vs. C groups. B: Graves’ disease, C: TAO. Table S10. The PPI analysis of the differentially expressed proteins in B vs. C groups. B: Graves’ disease, C: TAO. [file FEB4-11-1930-s001.zip › feb413172-sup-0002-FigS2.pdf]

Figure S2

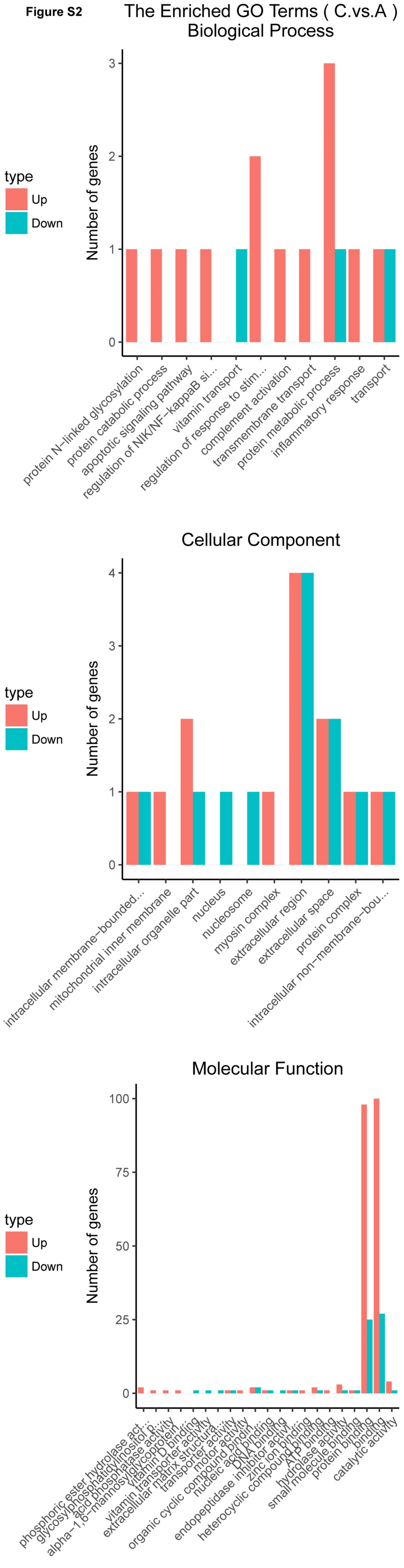

The Enriched GO Terms (C.vs.A)
Biological Process
Number of genes
0
1
2
3
type
Up
Down
protein N−linked glycosylation
protein catabolic process
apoptotic signaling pathway
regulation of NIK/NF−kappaB si...
vitamin transport
regulation of response to stim...
complement activation
transmembrane transport
protein metabolic process
inflammatory response
transport
Cellular Component
Number of genes
0
1
2
3
4
type
Up
Down
intracellular membrane−bounded...
mitochondrial inner membrane
intracellular organelle part
nucleus
nucleosome
myosin complex
extracellular region
extracellular space
protein complex
intracellular non−membrane−bou...
Molecular Function
Number of genes
0
25
50
75
100
type
Up
Down
phosphoric ester hydrolase act...
glycosylphosphatidylinositol p...
acid phosphatase activity
alpha−1,6−mannosylglycoprotein...
vitamin D binding
vitamin transporter activity
extracellular matrix structura...
transporter activity
motor activity
organic cyclic compound bindin...
nucleic acid binding
DNA binding
endopeptidase inhibitor activi...
zinc ion binding
heterocyclic compound binding
ATP binding
hydrolase activity
small molecule binding
protein binding
binding
catalytic activity
