# Supplementary figures and images for: Differentiation between thyroid‐associated orbitopathy and Graves’ disease by iTRAQ‐based quantitative proteomic analysis
Source: FEBS Open Bio. 2021 May 26;11(7):1930–40. doi: 10.1002/2211-5463.13172 (PMC8255837; doi:10.1002/2211-5463.13172)

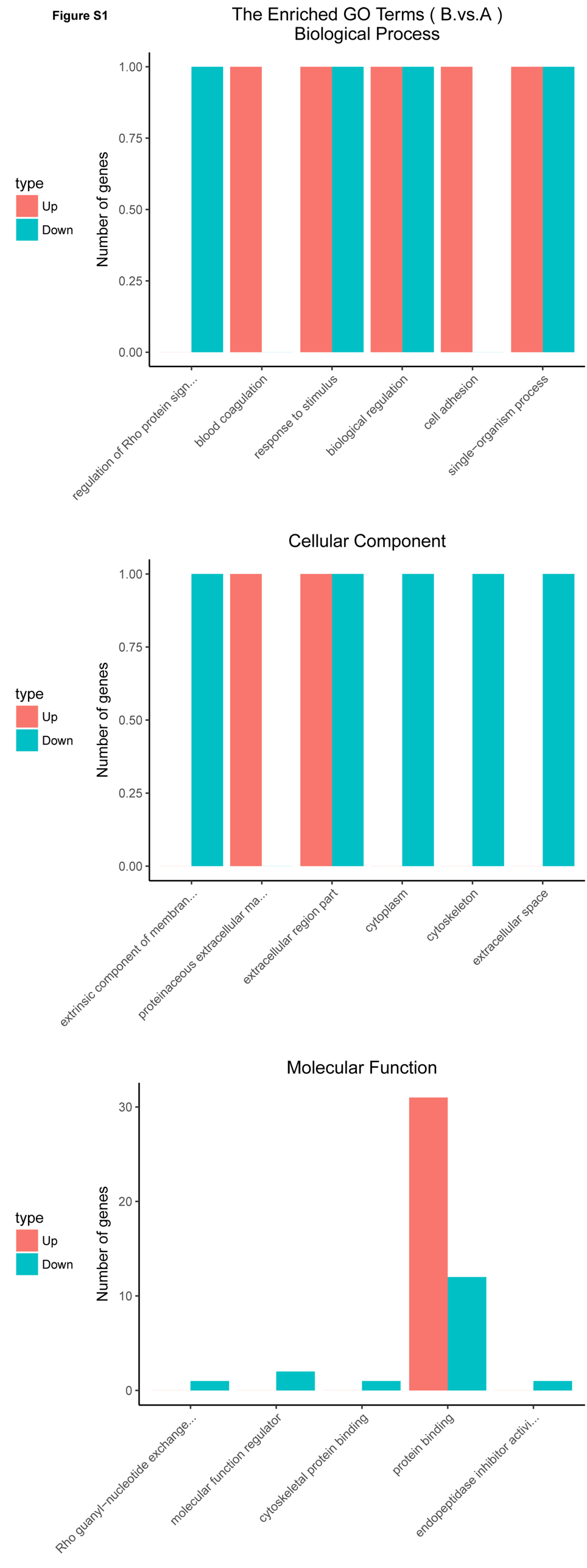

Supplement: Supplementary file 1 — Fig S1. The details of the GO enrichment analysis involving B vs. A groups. Fig S2. The details of the GO enrichment analysis involving C vs. A group. Table S1. Peptide fraction separation liquid chromatography elution gradient table. Table S2. Liquid chromatography elution gradient table. Table S3. All differentially expressed proteins identified by iTRAQ in A vs. B groups, A vs. C groups, and B vs. C groups. A: Healthy controls, B: Graves’ disease, C: TAO. Table S4. The differentially expressed proteins identified by iTRAQ in A vs. B groups. A: Healthy controls, B: Graves’ disease. Table S5. The differentially expressed proteins identified by iTRAQ in B vs. C groups. B: Graves’ disease, C: TAO. Table S6. The differentially expressed proteins identified by iTRAQ in A vs. C groups. A: Healthy controls, C: TAO. Table S7. The KEGG analysis of all differentially expressed proteins in A vs. B groups, A vs. C groups, and B vs. C groups. A: Healthy controls, B: Graves’ disease, C: TAO. Table S8. The KEGG analysis of the differentially expressed proteins in A vs. C groups. A: Healthy controls, C: TAO. Table S9. The KEGG analysis of the differentially expressed proteins in B vs. C groups. B: Graves’ disease, C: TAO. Table S10. The PPI analysis of the differentially expressed proteins in B vs. C groups. B: Graves’ disease, C: TAO. [file FEB4-11-1930-s001.zip › feb413172-sup-0001-FigS1.jpg]
